# Prenatal Diagnosis and Findings in Ureteropelvic Junction Type Hydronephrosis

**DOI:** 10.3389/fped.2020.00492

**Published:** 2020-09-04

**Authors:** Recep Has, Tugba Sarac Sivrikoz

**Affiliations:** Division of Perinatology, Department of Obstetrics and Gynecology, Istanbul Faculty of Medicine, Istanbul University, Istanbul, Turkey

**Keywords:** fetal hydronephrosis, ureteropelvic junction obstruction, fetal pelviectasia, pediatric urinary tract dilation, ultrasound

## Abstract

The widespread use of obstetric ultrasonography has increased the detection rate of antenatal hydronephrosis. Although most cases of antenatal hydronephrosis are transient, one third persists and becomes clinically important. Ultrasound has made differential diagnosis possible to some extent. Ureteropelvic junction type hydronephrosis (UPJHN) is one of the most common cause of persistent fetal hydronephrosis and occurs three times more in male fetuses. It is usually sporadic and unilateral. However, when bilateral kidneys are involved and presents with severe hydronephrosis, the prognosis may be poor. Typical ultrasound findings of UPJHN is hydronephrosis without hydroureter. The size and appearance of the fetal bladder is usually normal without thickening of the bladder wall. Several grading systems are developed and increasingly being used to define the severity of prenatal hydronephrosis and provides much more information about prediction of postnatal renal prognosis. If fetal urinary tract dilation is detected; laterality, severity of hydronephrosis, echogenicity of the kidneys, presence of ureter dilation should be assessed. Bladder volume and emptying, sex of the fetus, amniotic fluid volume, and presence of associated malformations should be evaluated. Particularly the ultrasonographic signs of renal dysplasia, such as increased renal parenchymal echogenicity, thinning of the renal cortex, the presence of cortical cysts, and co-existing oligohydramnios should be noticed. Unfortunately, there is no reliable predictor of renal function in UPJHN cases. Unilateral hydronephrosis cases suggesting UPJHN are mostly followed up conservatively. However, the cases with bilateral involvement are still difficult to manage. Timing of delivery is also controversial.

## Key Concepts

- UPJHN is the most common cause of persistent antenatal hydronephrosis. It is usually unilateral and three times more in male fetuses.
- UPJHN may be 10–30% bilateral and should be managed cautiously for the deterioration of renal functions.
- In all cases with prenatal UPJHN, AP renal pelvis diameter, presence and localization of calyx dilation, renal parenchymal features, presence of urinoma and oligohydramnios should be assessed.
- Patients in the high-risk group should be monitored during the prenatal period with an interval of 2–4 weeks, however patient monitoring should be customized according to the other negative findings.
- When UPJHN is detected during the prenatal period, consulting with pediatric urologists before delivery may contribute the postnatal management plans.

## Introduction

Congenital hydronephrosis is one of the most common anomalies encountered at the prenatal ultrasound evaluation. It is observed in 1–4% of all pregnancies (1, 2).

Prenatal urinary system evaluation should preferably follow an anatomical sequence in order to identify the cause of the dilation. Therefore, urinary system examination in the prenatal period should demonstrate position of bilateral kidneys, dilation of renal pelvis and presence of calyx dilation (central and peripheral), echogenicity of kidney parenchyma, both ureters, bladder size and wall thickness, and anatomy of the external genitalia.

Detection of urinary system malformations are related to the week of gestation when the screening has been performed. Urinary system is usually assessed at 19–21 weeks of gestation, and late onset hydronephrosis is commonly missed during this period. Therefore, urinary system anomalies are not infrequently identified in the third trimester up to a rate of 5% (3).

## Urinary System Evaluation With Prenatal Ultrasound

**Figure 1:**
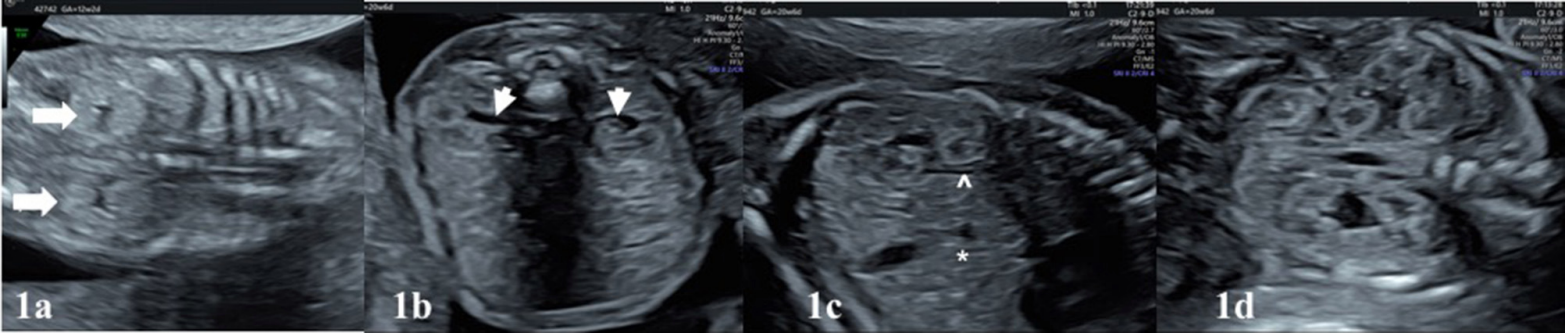
In **(a)**, kidneys of a 12-week fetus. The best visualization of kidneys (white bold arrows) can be obtained in coronal plane with transvaginal ultrasound at this gestational age. **(b)** Transabdominal ultrasound shows the normal appearance of both kidneys on the axial plane at 20 + 6 weeks of gestation. Kidneys are located both sides of vertebra and renal pelvis (white arrow-heads) oriented toward the midline. **(c)** Longitudinal plane, echogenicity of the kidney is comparable to the liver (*). Hypoechoic adrenal gland (∧) is located cranial to the kidney. Cortico-medullary differantiation (white chevron) can be noticed. **(d)** Coronal plane shows two bean-like kidneys in the same section. This plane is useful to compare the kidneys. The corticomedullary differantiation can be noticed easily on the coronal plane.

**Figure 2:**
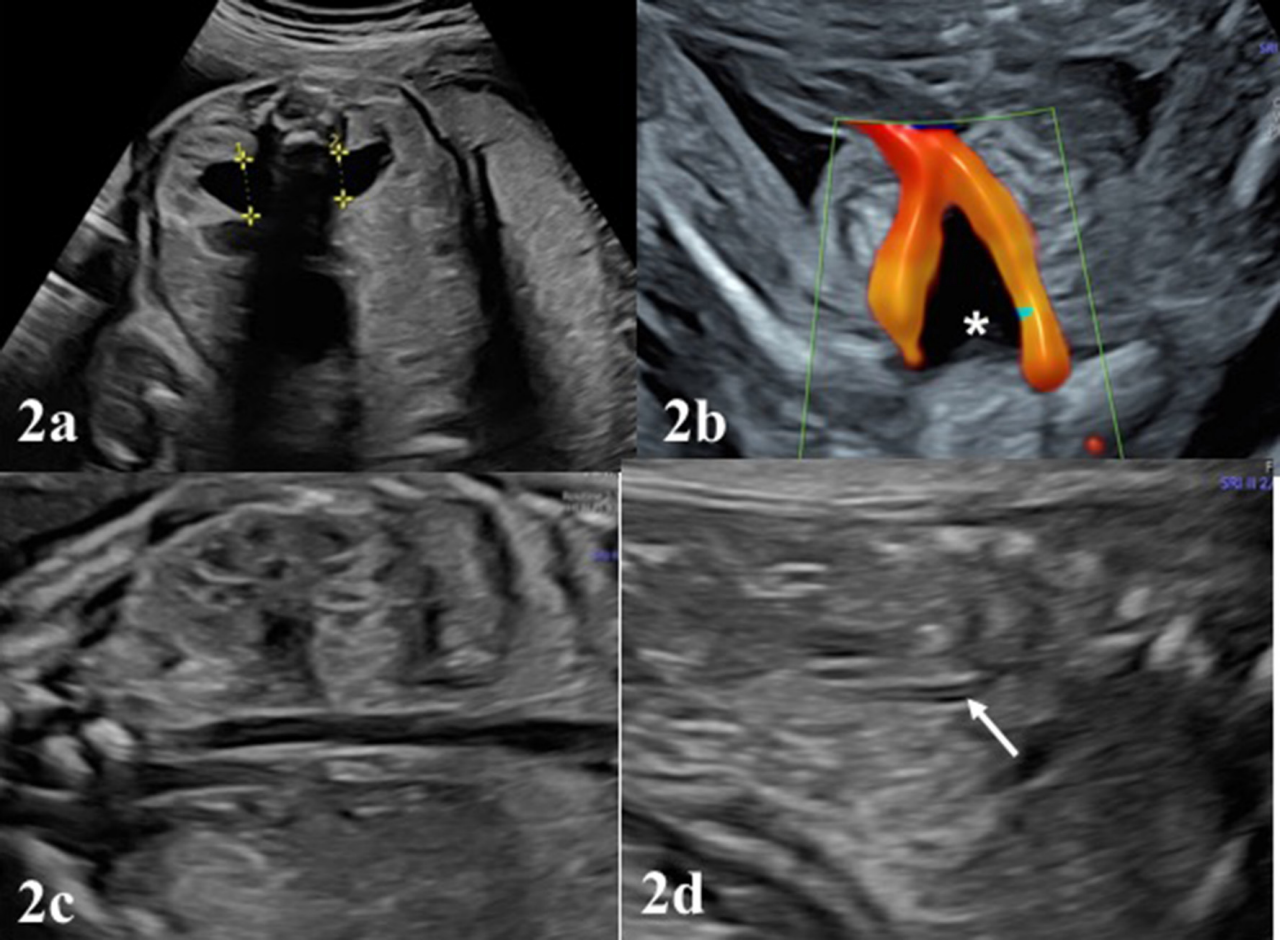
**(a)**, the normal appearance of the fetal bladder (white asterisk) in the pelvis between both umbilical vessels. **(b)** The appearance of kidneys in the 22 weeks with mild renal pelvis diameter. Antero-posterior diameter (APD) of pelvis renalis should be measured in the axial plane, better if fetus in dorso-anterior position. **(c)** Note the corticomedullary differentiation in a normal appearing fetal kidney. In **(d)**, white arrow depicts the surrenal gland lying on the kideny, shown as echogenic medulla and hypoechogenic cortex.

Examination of the urinary system in fetal ultrasound scan begins with identifying the presence of kidneys and bladder. From the 11–12 weeks of gestation, fetal kidneys can be visualized by transvaginal ultrasonography as hyperechogenic structures (4) (Figure 1a). Fetal kidneys are imaged in the abdomen at both sides of vertebral column on axial, longitudinal and coronal planes (Figures 1b–d). Kidneys appear as two round paravertebral structures on axial views and renal pelvis oriented toward the midline (Figure 1b). The appearance of normal kidney looks bean-like on longitudinal and coronal planes. At coronal plane, both kidneys can be visualized on the same section and can be compared to each other (Figure 1d). Size of the kidneys can be evaluated by measuring the renal length and comparing it to normal charts. Normal kidneys have the same echogenicity with liver and spleen (Figure 1c). When kidney echogenicity is higher than spleen or liver, it is considered to be hyperechogenic. The cortex and medulla of the kidneys also become differentiable in fetuses older than 18 weeks and difference becomes more significant toward the third trimester (Figures 1c,d, 2c). The renal cortex is slightly echogenic at the periphery of medulla. The adrenal glands are located cranial to the kidneys as more hypoechogenic structures (Figures 1c, 2d).

After evaluation of the location of both kidneys, parenchymal features, the assessment of the dilation (pelviectasis) of the renal pelvis should be made. From the beginning of the second trimester, renal pelvis becomes detectable and the kidneys generally lose their previous hyperechogenic appearance. Renal pelvis always appears as a sonolucent area in the medial of the kidneys (Figure 1b). Pelviectasis or hydronephrosis is evaluated in the sections of the fetal abdominal transverse planes, by measuring the anteroposterior diameter (APD) of the renal pelvis, where possible the fetal back is perpendicular to the probe (Figure 2a). Dilation of the renal pelvis may differ by gestational week, maternal hydration, or bladder distension (5–7).

The bladder can be visualized into the fetal pelvis from the 10th week of pregnancy, however from the 12th week on the pelvis it should be visible as a sonolucent cystic structure between both umbilical vessels (Figure 2b). Ideal position to measure bladder wall thickness is near the umbilical arteries in axial plane of the fetal pelvic area. Bladder wall thickness does not exceed 2 mm in prenatal period regardless of gestational week (8, 9). Fetal bladder empties and refills every 25–30 min during second and third trimester. Although nomogram charts to check the bladder size may be used, subjective assessment is usually gives satisfactory information. Ureters and urethra are not typically visible structures in the prenatal period. These structures may be visualized when dilated in case of bladder outlet obstruction or vesicoureteral reflux.

Fetal urine is the primary source of the amniotic fluid after 14–16 weeks of gestation. Normal volume of amniotic fluid is not only the predictor of normal renal function, but also needed for proper development of fetal lungs. Therefore, the assessment of urinary system should also include evaluation of amniotic fluid volume.

To summarize, when antenatal hydronephrosis (ANH) is diagnosed, the following parameters should be examined in a certain order by ultrasound:

- Severity and progress of hydronephrosis: As the APD increases, the possibility of concomitant congenital urinary system anomalies increases. Presence of calyx dilation and involvement of central or peripheral calices should be assessed. Repeat ultrasound examinations in the second and third trimesters will guide to determine neonatal prognosis. In the presence of severe pelviectasis, the need for surgical intervention may significantly increase in the neonatal period (10).
- Laterality: Such as, if UPJHN is bilateral, the risk for additional congenital kidney anomalies and renal function impairment is increased.
- Parenchymal appearance: An echogenic renal cortex suggests abnormal change of the renal parenchyma. The presence of parenchyma thinning or cortical cyst is associated with impaired renal function. These changes are often observed as consequence of UPJHN, and other lower urinary tract obstructions such as posterior urethral valve (PUV) or VUR.
- Urinoma/urinary ascites: Urinoma is an encapsulated paranephric pseudocyst confined to the Gerota's fascia. It often develops secondary to obstructive pathologies. Although it is rare, it co-exists with dysplastic non-functional kidney on the same side in 80% of cases (11). Urinary ascites develops in cases with lower urinary tract obstruction following to spontaneous or iatrogenic rupture of the kidney or bladder.
- Ureter: Ureter dilation is not observed with UPJHN. It is typically associated with obstructions distal to the uretero-pelvic junction, such as PUV and other infravesical obstructions or vesico-ureteral reflux (VUR) (**Figures 4a,b**).
- Bladder/ureterocele: Bladder size, appearance, and wall thickness are normal in UPJHN. If increased bladder wall thickness and trabeculation is detected, obstruction distal to the bladder neck (PUV) should be considered (**Figures 4a,b**). Ureterocele is a cystic dilation of ureter detected inside the bladder. It is usually seen with duplication of the collecting system anomalies, secondary to distended ureters.
- Amniotic fluid volume: Oligohydramniosis develops after decreased urine output due to the urinary tract obstruction or decreased urine production as a result of impaired renal function. It is usually predictor of poor prognosis and implicates severe renal disease, where both kidneys are affected.

## Evaluation of Anteroposterior Pelvis Diameter (APD) and Classification

Fetal hydronephrosis is usually detected by ultrasound in the second trimester and defined as a renal pelvis diameter measurement above ≥4 mm. As gestational week progresses, definition of threshold values for dilation of the renal pelvis increases in the prenatal period (7) (Table 1). A measurement of ≤ 3 mm is considered normal in all gestational weeks (12). Mild hydronephrosis (APD 4–10 mm) may be a transient finding, or rarely associated with renal or chromosomal abnormalities. More severe dilation increases the risk of congenital anomalies of the kidney and urinary tract (CAKUT). Nguyen et al. reported that 50–70% of the urinary system dilations detected in antenatal period are temporary (7). Determining specific limit values for each trimester of the pregnancy is important for the frequency, follow-up and management of the pelviectasis in both prenatal and postnatal period. Among the prenatal mild pelviectasis cases, only a small proportion have a serious problem in the postnatal period. Renal pathology is confirmed in postnatal period in 12–14% of mild, 45% of moderate and 90% of severe pelviectasis cases detected in the second and third trimesters of pregnancy (13). Presence of calyx dilation and identification of parenchymal echogenicity is important for the prediction of clinically significant ANH cases (14). When calyx dilation (pelvicaliectasis) is accompanied to the renal pelvis dilation, the location should also be defined as central or peripheral. This is important for the classification and prediction of prognosis.

**Table 1:** Stage of antenatal hydronephrosis (ANH) based on renal pelvis APD in relation to gestational age.

**Degree of ANH**	**APD at 2nd trimester**	**APD at 3rd trimester**
Mild	4–7 mm	7–9 mm
Moderate	7–10 mm	9–15 mm
Severe	>10 mm	>15 mm

In order to plan postnatal follow-up, several classification systems for the urinary tract dilation (UTD) have been proposed based on ultrasonographic findings. The most common used classification systems are the Society for Fetal Urology (SFU) Hydronephrosis Grading System and the Urinary Tract Dilation (UTD) Classification System (1, 7). SFU system has five grades (0–4) (7). In this classification system, intra and extra-renal dilation of the renal pelvis is defined subjectively. Dilation of central and peripheral calyces is assessed and parenchymal thickness is described subjectively (Table 2).

**Table 2:** The sonographic SFU Grading system for fetal urology (https://www.uab.edu/images/peduro/SFU).

	**Pattern of renal sinus**
SFU grade 0	No splitting
SFU grade 1	Urine in pelvis barely splits sinus
SFU grade 2	Urine fills intrarenal pelvis
SFU grade 2	Urine fills extrarenal pelvis, major (central) calyces dilated
SFU grade 3	SFU G2 and minor (peripheral) calyces uniformly dilated and parenchyma preserved
SFU grade 4	SFU G3 and parenchyma thin

In UTD classification system, measurement of renal pelvis antero-posterior diameter, presence of calyx dilation, subjective definition of parenchymal thickness and parenchymal appearance are specified. Dilation of ureters, assessment of bladder (wall thickness, ureterocele, and dilated posterior urethra) and presence of oligohydramnios are also considered (Table 3) (1). The most important difference of this classification is, quantitative assessment of urinary system (1). Patients are monitored in the prenatal period by separating them into low-risk and high-risk groups according to the severity of the features determined in the UTD classification. As the grade advances prognostic significance increases in UTD system. For example, a fetus has 9 mm renal pelvis AP diameter with increased echogenicity of the kidneys is classified in high-risk group. UTD system may be used also for postnatal cases. This system can evaluate antenatal and postnatal hydronephrosis simultanously, therefore, some studies stated that it is the classification system with the highest correlation with neonatal results (15).

**Table 3:** Urinary Tract Dilation Classification System.

**Ultrasound findings**	**Time at presentation**		
	16–27 weeks	≥28 weeks	Postnatal (>48 h)
Anterior-posterior renal pelvis diameter (APRPD)	<4 mm	<7 mm	<10 mm
Calyceal dilatation Central Peripheral	No No (if yes high risk)	No No (if yes high risk)	No No (if yes high risk)
Parenchymal thickness	Normal	Normal	Normal
Parenchymal appearance(echogenicity, corticomedullar differentiation, pericortical cysts, urinoma)	Normal	Normal	Normal
Ureter(s)	Normal	Normal	Normal
Bladder	Normal	Normal	Normal
Oligohydramnios	No	No	NA

An alternative classification system for primary UPJHN was proposed by Onen in 2007 (14). According to Onen's grading system (AGS), isolated pelvic dilation was classified as grade 1. Grade 2 is the presence of calyx dilation in addition to renal pelvis dilation. Grade 3 includes <50% loss in renal parenchyma and grade 4 has severe parenchymal loss. AGS covers essentially neonatal period and is used only for primary UPJHN. Using AGS grading system may simplify the follow-up and treatment plan in postnatal patients with UPJHN. However, it may be difficult to assess the thinning of medulla and cortex separately in the fetus particularly before the third trimester and this system needs to be studied in prenatal period to make a statement on it. UTD system is actually combination of APD classification and SFU and provides more information for the diagnosis. More studies are needed to be done in the prenatal period to compare the SFU, UTD, and AGS classification systems for UPJHN. A comparison of all three grading systems and radiological assessment is demonstrated in Table 4 (16).

**Table 4:** Prenatal and postnatal evaluation systems used in UPJHN classification (Courtesy of Onen A, 2020, in press).

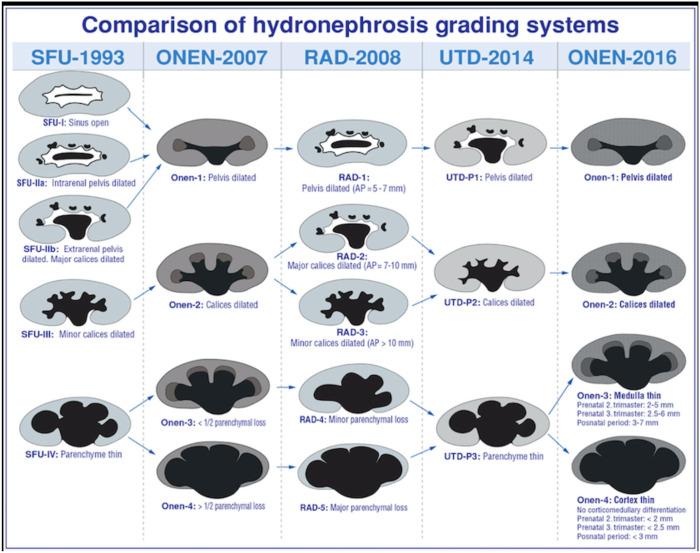

## Ultrasonographic Findings of Upjhn in the Prenatal Period

The main cause of hydronephrosis is obstruction at any level of the urinary system. Some obstructive changes may develop very early in fetal life and may cause cystic-dysplastic pathology in the fetal kidney. Therefore, the initial time of obstruction and its consequences are as important as the severity of the dilation. UPJHN is the most common reason of ANH Other causes include vesico-ureteral reflux, uretero-vesical junction obstruction, posterior urethral valve, and other rare incidents (1). Each is caused by different levels of obstruction and carries different ultrasonographic features. Accurate prenatal diagnosis will not only provide appropriate follow-up and prenatal interventions, but also help to prepare for the postnatal management.

The most common pathological cause of antenatal hydronephrosis UPJHN constitutes 10–30% of antenatal hydronephrosis (7). It is reported in 1/750–1/1,500 live births (17). UPJHN is three times more common in males than in females particularly in the neonatal period (18). It is usually sporadic, unilateral and mostly the left side (68%) is affected (19, 20). The etiology of uretero-pelvic junction obstruction is obscure with an adynamic narrow segment causing the obstruction (21).

**Figure 3:**
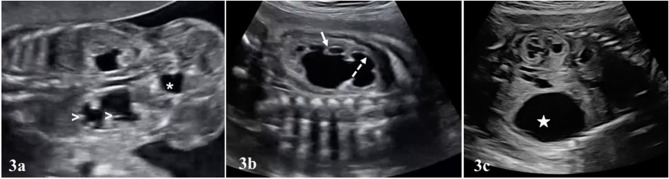
In **(a)**, bilateral UPJHN is seen in a fetus at 22 weeks of pregnancy. Renal pelvis dilated in both kidneys. There is also dilated calyces in the lower kidney (white arrow heads). The size and appearence of the bladder (asterisk *) is normal, and ureters are not visible. **(b)** Central (dashed arrow) and peripheral calyces (short arrow) in the renal pelvis are dilated. Renal parenchyma is also thinned. **(c)** Increased echogenicity in the renal parenchyma (upper kidney). A large urinoma (white star) was seen in the lower kidney.

Typical finding in UPJHN in prenatal ultrasound is unilateral renal pelvis dilation with or without caliectasis, while the ureter is not dilated (Figures 3a,b, Video S1). Bladder dimensions and bladder wall thickness are normal in UPJHN. Presence and localization of caliectasis as central or peripheral, is important for grading systems of SFU and UTD, particularly in prenatal period. Evaluation of appearance of the parenchyma is also essential. As the pelviectasia/caliectasia advances, the parenchyma thickness decreases and echogenicity increases on the affected kidney.

Assessment of the severity of the dilation is essential for the grading systems in all cases of hydronephrosis detected in prenatal ultrasound. For example, in SFU grade 3 dilation, pelvis and peripheral calyces are dilated, but parenchymal thickness is normal (Table 2) (7). However, in SFU grade 4, the parenchyma gets thinner. The difference between SFU grade 1 and 2 is the presence of central calyx dilation in grade 2, independent of the measurement of pelvic dilation. Similar to the UTD system, the SFU grading system can be used both in the pediatric and in the prenatal period.

Most of the urinary system anomalies can be diagnosed by prenatal ultrasound. However, with maternal obesity, advanced gestational week or presence of oligohydramniosis, visualization of the structures may be challenging. Fetal magnetic resonance imaging (MRI) is an important adjunct to ultrasound in evaluation of fetal urogenital system. While ultrasound remains the primary diagnostic modality, MRI helps in more complicated cases or where ultrasound is limited due to technical factors such as poor acoustic window (22). Prenatal MRI may also be useful in differential diagnosis of VUR from UPJHN, particularly in positions where the fetal pelvis is difficult to visualize the ureter dilation (23). Imaging in T1-T2-weighted MRI sequences is rather guiding to evaluate the functional status of fetal kidneys (24). Kajbafzadeh et al. reported that the sensitivity of prenatal MRI in differential diagnosis of urinary system anomalies was 92% in their study (24). Particularly, MRI is informative when type of calyx dilation is difficult to distinguish in cases with prenatal UPJHN (24).

## Other Urinary System Ultrasound Findings in Prenatal Upjhn

**Figure 4:**
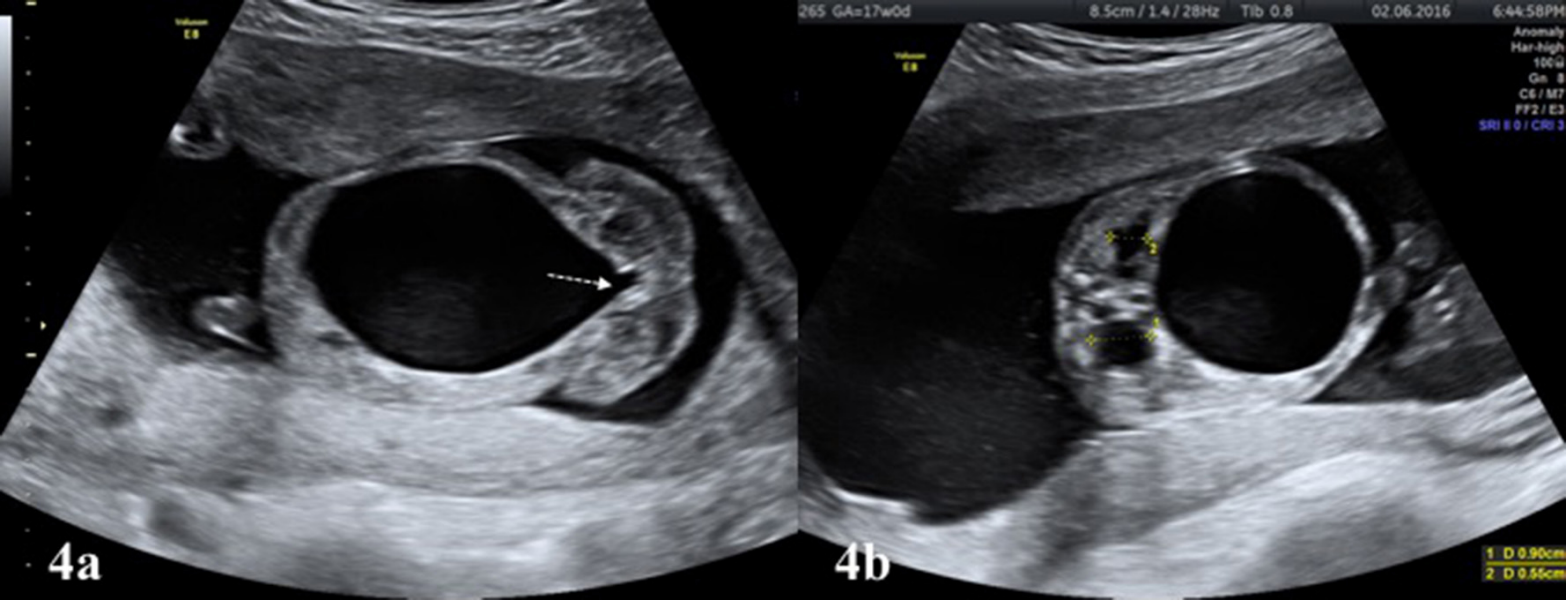
**(a)** Large bladder with “key hole” appearance (white colored dashed arrow) is typical finding of infravesical obstruction, mostly due to PUV in male fetuses. **(b)** The affected kidneys in the same fetus **(a)** showing increased renal pelvis diameter and parenchymal echogenicity.

The excessively dilated collection system rarely ruptures spontaneously in UPJHN. As a result, an encapsulated paranephric pseudocyst (urinoma) confined to the Gerota's fascia is formed (25, 26). The urinoma is located on the affected kidney side as an elliptical or crescentric cystic mass adjacent to the kidney and vertebral column (Figure 3c). Similar to in UPJHN, urinoma can occur in the cases with PUV, where the intrarenal pressure can be high enough to cause rupture. PUV can be differentiated more easily with the presence of large bladder which typically looks like a key hole and coexist with bilateral hydroureteronephrosis (Figures 4a,b).

Urinoma is often detected at 19–30 weeks of gestation in UPJHN. Other cystic structures such as lymphangioma, neuroblastoma and ureteric duplication, which are located in this region, should also be considered in differential diagnosis (27). Modifying the time of delivery or interventions such as shunt placement to the urinoma is not necessary in the prenatal period. Urinomas may regress spontaneously before birth, but this does not imply better prognosis. The presence of urinoma with the dysplastic changes in the kidney parenchyma is associated with a poor prognosis (25, 27). Postnatal normal kidney function in UPJHN cases affected by ipsilateral urinoma is only 7% (28, 29). Another study reported that the prognosis is more morbid in the presence of urinoma with prenatal UPJHN than other urinary tract obstructions (11).

Oligohydramniosis is one of the most important prognostic parameters in evaluating kidney functions in fetal life. Single vertical pocket (SVP) or amniotic fluid index (AFI) are the most frequently used methods in the evaluation of oligohydramniosis. The threshold used to define oligohydramniosis is SVP ≤2 cm or AFI ≤ 5 cm. Bilateral UPJHN with dysplastic changes in kidneys and subsequent oligohydramniosis indicates poor prognosis (18, 30). Since chronic oligohydramniosis is associated with fetal lung hypoplasia, it affects neonatal prognosis directly.

Bilateral UPJHN is detected 10–30% in prenatal ultrasound (20) (Figure 3a, Video S1) and it is frequently detected in <6 month old infants in neonatal period (8, 20). The most diagnostic challenge is differentiation of bilateral UPJHN cases with VUR. VUR is more common in girls, and hydronephrosis is typically presented with ureter dilation (Video S2). Since postnatal management of VUR cases is different from UPJHN, its differentiation in the prenatal period is also important for follow up and management.

**Figure 5:**
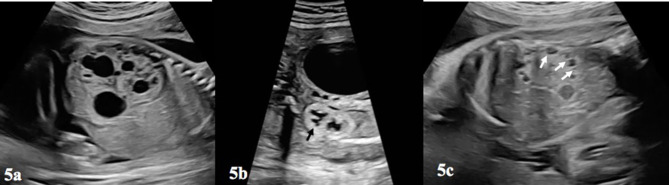
**(a)** Depicts a multicystic-dysplastic kidney, which can be seen in the contralateral kidney in a fetus with prenatal UPJHN in other kidney. Notice the difference between **(a,c)** and Figure 3. **(b)** This figure shows increased echogenicity of the lower kidney and loss of cortico-medullary differentiation of renal parenchyma (blac arrow). **(c)** Pericortical cysts (white arrows) and echogenic parenchyma was shown on **(b)**.

Another factor determining the prognosis in prenatal and postnatal period is the appearance of the contralateral kidney. Additional urinary system anomalies are present in 50% of UPJHN. The most common condition which is seen in contralateral kidney is UPJHN. Among the other urinary system anomalies, multicystic dyplastic kidney (MCDK) (Figure 5a), VUR, duplication of the collecting system, rotation and fusion anomalies in the other kidney are reported in conjunction with UPJHN (18). The actual incidence of MCDK with UPJHN is unknown, and its frequency has been reported to range between 2 and 27% (31). Since monitoring and management changes in the presence of other kidney anomalies, the anatomy and location of the contralateral kidney should be carefully evaluated.

## Other System Anomalies Co-existing With Upjhn in Prenatal Ultrasonography

The incidence of chromosome anomalies accompanying prenatal UPJHN obstruction is relatively low and reported around 1–3%. Karyotype analysis is not crucial in isolated cases when other parameters are favorable. However, in the presence of associated anomalies, prenatal diagnostic invasive procedures should be offered (32). Congenital heart disease, VA(C)TER(L) association, Schinzel-Giedon syndrome and Camptomelic dysplasia are among the most common other system anomalies associated with UPJHN in the prenatal period (33, 34). A comprehensive fetal anatomy scan should be carried out for other systems, particularly including fetal heart, gastrointestinal tract and spine (8, 18).

## Parameters Determining Poor Prognosis in Prenatal Upjhn

Prenatal management of UPJHN primarily depends on the APD, taken into account by the gestational week. Progression of the obstruction, presence of dilation in calyx system and parenchymal condition of the affected kidney guides the follow-up. Gestational age at presentation, presence of unilateral or bilateral involvement, and other coexisting anomalies are important to determine the prognosis. If there is bilateral UPJ obstruction, associated anomaly in the contra-lateral kidney and/or oligohydramniosis, the prognosis will be negatively affected.

Jiang et al. reported a spontaneous regression rate of 61%, and persistence rate of 23% in cases diagnosed with antenatal bilateral UPJHN (35). Probability of postnatal surgery was 15% in cases where renal pelvis AP diameter was ≥15 mm (35). When calyx dilatation is ≥10 mm, spontaneous resolution is 37%, the possibility of persistence is 29% and the surgical requirement is around 33% (35). However, in cases where calyx dilatation was <5 mm and AP diameter was <10 mm, 90–100% regression was reported, and there is virtually no need for surgery (0–3.7%) (35). This study has shown that pelvic AP diameter plays a primary role along with calyx dilation in determining the follow-up process and the need for intervention. Perlman et.al. analyzed the outcome of 35 fetuses diagnosed with severe isolated hydronephrosis (AP diameter >15 mm) and 48 fetuses with associated with congenital anomalies of the kidney and urinary tract (CAKUT) (10). The CAKUT group was associated with a significantly increased incidence of postnatal need for surgery (17.6 vs. 44.2%, *P* = 0.014), dysplastic kidney (0 vs. 14%, *P* = 0.023), and total abnormal outcome (52.9 vs. 86%, *P* = 0.001). A recent meta-analysis assessed the diagnostic value of APD of the fetal renal pelvis in predicting postnatal surgery. Diagnostic OR of antero-posterior diameter for predicting postnatal surgery was 13.3 mm. The authors suggested 15 mm AP diameter of APD may be used as a cut-off for the prediction of surgery (36). Elmaci et al. emphasized that spontaneous resolution rate was 71%, especially in cases with UPJHN-type antenatal hydronephrosis, where APD was ≤ 20 mm (37).

Regardless of etiology of hydronephrosis, abnormal parenchyma (thin and/or echogenic) appearance is a common parameter used in both SFU (grade 4) and UTD (high risk) classifications. The thickness and echogenicity of parenchyma affected by UPJHN is particularly important to predict renal function (Figures 3c, 5b). However, there is no consensus regarding the location of the assessment of parenchymal thickness prenatally. Moreover, subjective determination of parenchymal thickness may cause more conflicting results. Correlation between parenchymal thickness and prognosis is not clear even in postnatal studies (38). Nevertheless, loss of uniform structure of the renal parenchyma, presence of peri-cortical renal cysts (Figure 5c) and increased renal parenchymal echogenicity in prenatal ultrasound are associated with impaired renal function (Figures 3b, 5b) (39).

Despite all efforts, the contribution of SFU and UTD systems to prediction of prognosis in antenatal hydronephrosis is still uncertain (40). Both is proposed to be used regardless of etiology of hydronephrosis. Several studies have shown that inter-observer reliability of UTD classification is superior to SFU classification (41, 42). The use of other urinary system ultrasound parameters (kidney echogenicity, ureter dilation, ureterocele, oligohydramnios) in UTD classification increases its reliability. Renal pelvis AP diameter is the only quantitative criteria in UTD classification. However, other studies have shown that AP diameter does not make any significant predictive impact in terms of prognosis compared to other parameters (40, 41). A comparison of UTD and SFU grading system for their ability to predict time to hydronephrosis resolution showed that cumulative resolution rate at 3 years was higher in SFU grades (43). Among 401 patients 328 (82%) had resolution in 24 ± 18 months in study population (43). The lower the grade the better the resolution in both grading systems.

## Prenatal Follow-up and Management of Delivery

Fetuses with UPJHN should be followed up with ultrasound at regular intervals in prenatal period. Observation of regression, stable continuation or progression should be noted. Spontaneous resolution is often associated with mild dilated renal pelvis AP diameter. Of the 80% cases of the dilation between 4 and 8 mm are resolved, whereas only <15% of the >9 mm cases are regressed in the second trimester (44). Low-risk group includes patients with mild APD with normal kidney echogenicity, normal cortico-medullary differentiation, absence of peripheral calyx dilation. Therefore, it may be appropriate to re-evaluate the low-risk cases only for a second time in the third trimester. The unfavorable prognostic findings are; severe AP dilation (≥7 mm before 28 week or ≥10 mm after), increased kidney echogenicity, parenchymal thinning, peripheral calyx dilatation, presence of oligohydramnios, abnormality in the contra-lateral kidney and presence of bilateral UPJHN. Prenatal ultrasound follow-up examination in 2 week intervals is recommended by most authors in cases with unilateral severe UPJHN, bilateral UPJHN or contra-lateral kidney anomaly (7, 30). Other mild cases should be followed up with 4–6 week intervals until birth (1, 7, 45).

It is recommended to evaluate these cases together with pediatric urologists during the prenatal period, where possible. Multidisciplinary management of the cases will contribute positively to the postnatal outcome (46).

Several studies have shown that the timing or type of delivery does not affect the postnatal course in cases with UPJHN. Benjamin et al. investigated the impact of gestational age on urologic outcomes for the fetuses with hydronephrosis and concluded that late preterm/early term delivery resulted in worse short-term postnatal renal outcomes. They recommended delivery at 39 weeks (47). However, the cases with oligohydramniosis (bilateral UPJHN or contralateral kidney anomaly) are associated with loss of renal function in the third trimester, and earlier delivery may be considered for this group, although the benefit is questionable.

## Conclusion

Ultrasonography has the essential place in prenatal diagnosis, and has a key role in the antenatal diagnosis of kidney anomalies. Hydronephrosis is the most frequently diagnosed urinary system anomaly in the prenatal period. UPJHN is the most common pathological finding of the fetal genitourinary system. Although it is usually unilateral and have a favorable postnatal prognosis, outcome may be poor when bilateral or when associated with severity indicators. Unfavorable prognostic factors which indicate loss of kidney function are; increase in kidney echogenicity, loss of cortico-medullary differentiation, presence of pericortical cysts, and oligohydramniosis. Ultrasound follow up of the findings in the urinary system at certain intervals is important for the management of pre and postnatal period. Adaption of one of the classification systems such as UTD or SFU or AGS may contribute to the objective assessment of both prenatal and postnatal management. In the absence of obstetric risk factors such as presence of oligohydramnios, positive contribution of delivery timing to the prognosis has not been demonstrated yet.

## Author Contributions

RH: drafting the work and revising it. TS: providing images, drafting the work, and revising it. All authors listed on manuscript have participated in the present work.

## Conflict of Interest

The authors declare that the research was conducted in the absence of any commercial or financial relationships that could be construed as a potential conflict of interest.
