# Supplementary figures and images for: Prenatal Diagnosis and Findings in Ureteropelvic Junction Type Hydronephrosis
Source: Front Pediatr. 2020 Sep 4;8:492. doi: 10.3389/fped.2020.00492 (PMC7498649; doi:10.3389/fped.2020.00492)

## Slide 1
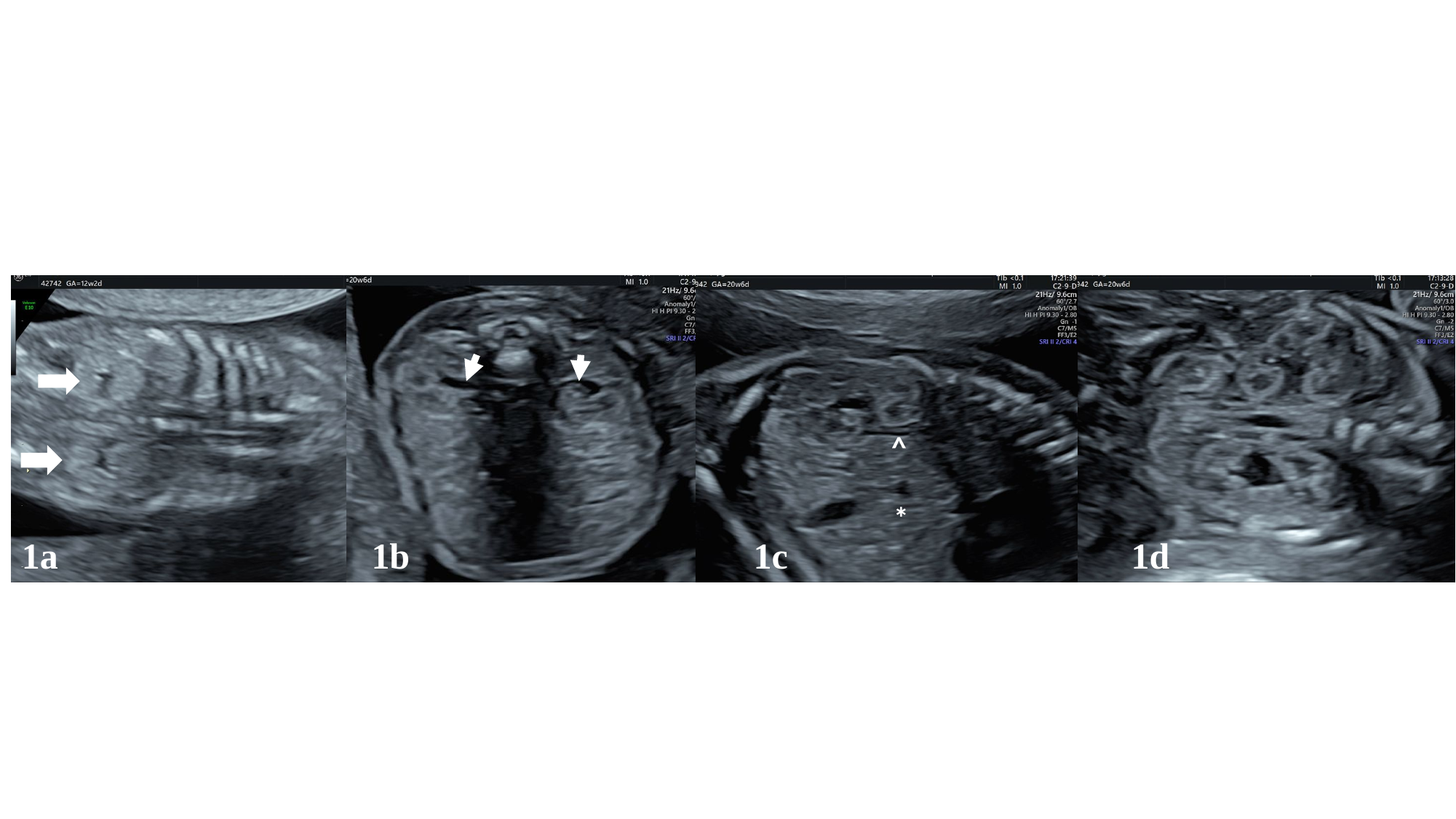

^
*
1c
1d
1b
1a
1a

## Slide 2
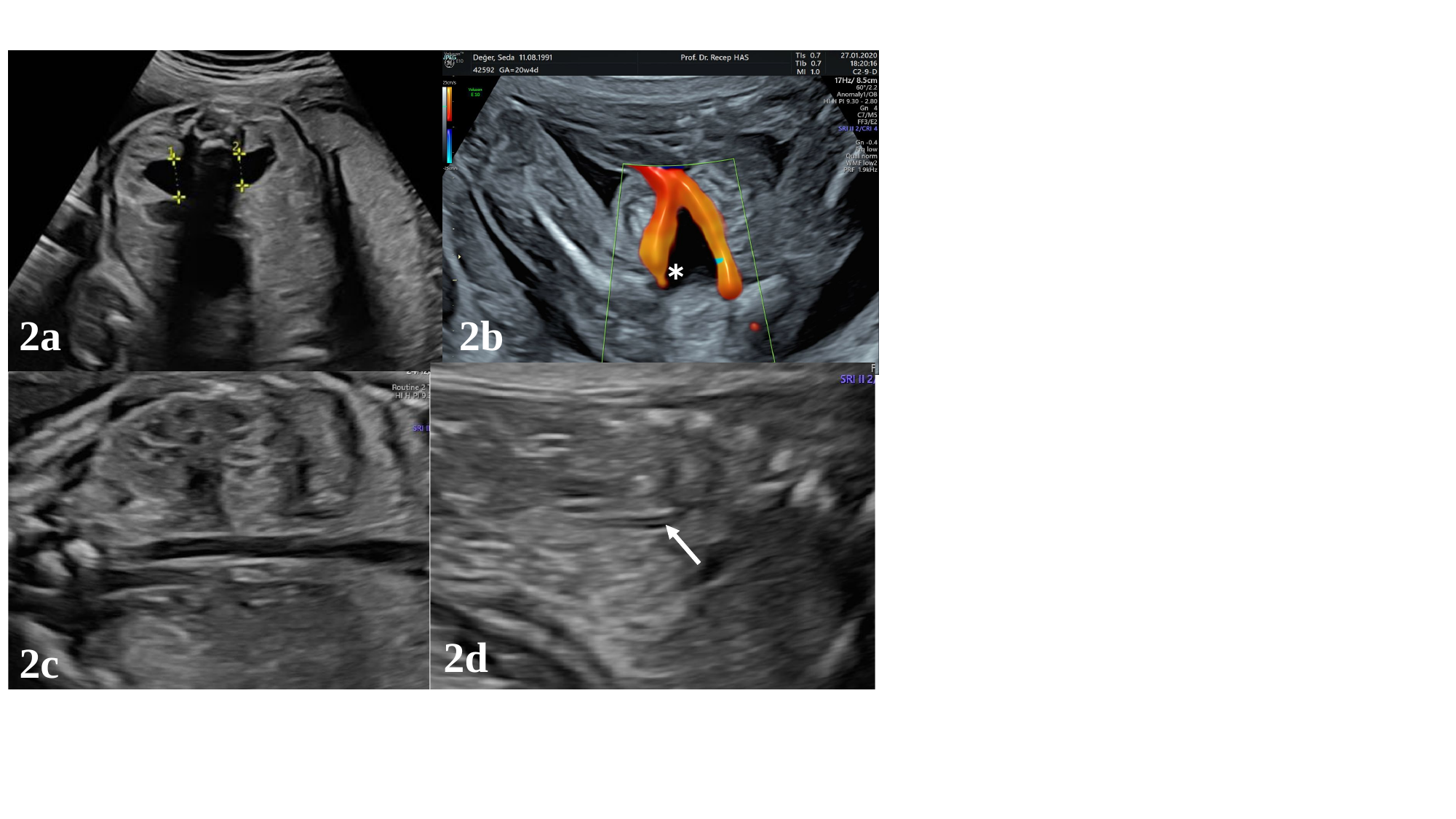

*
2a
2b
*
*
2b
2d
2c

## Slide 3
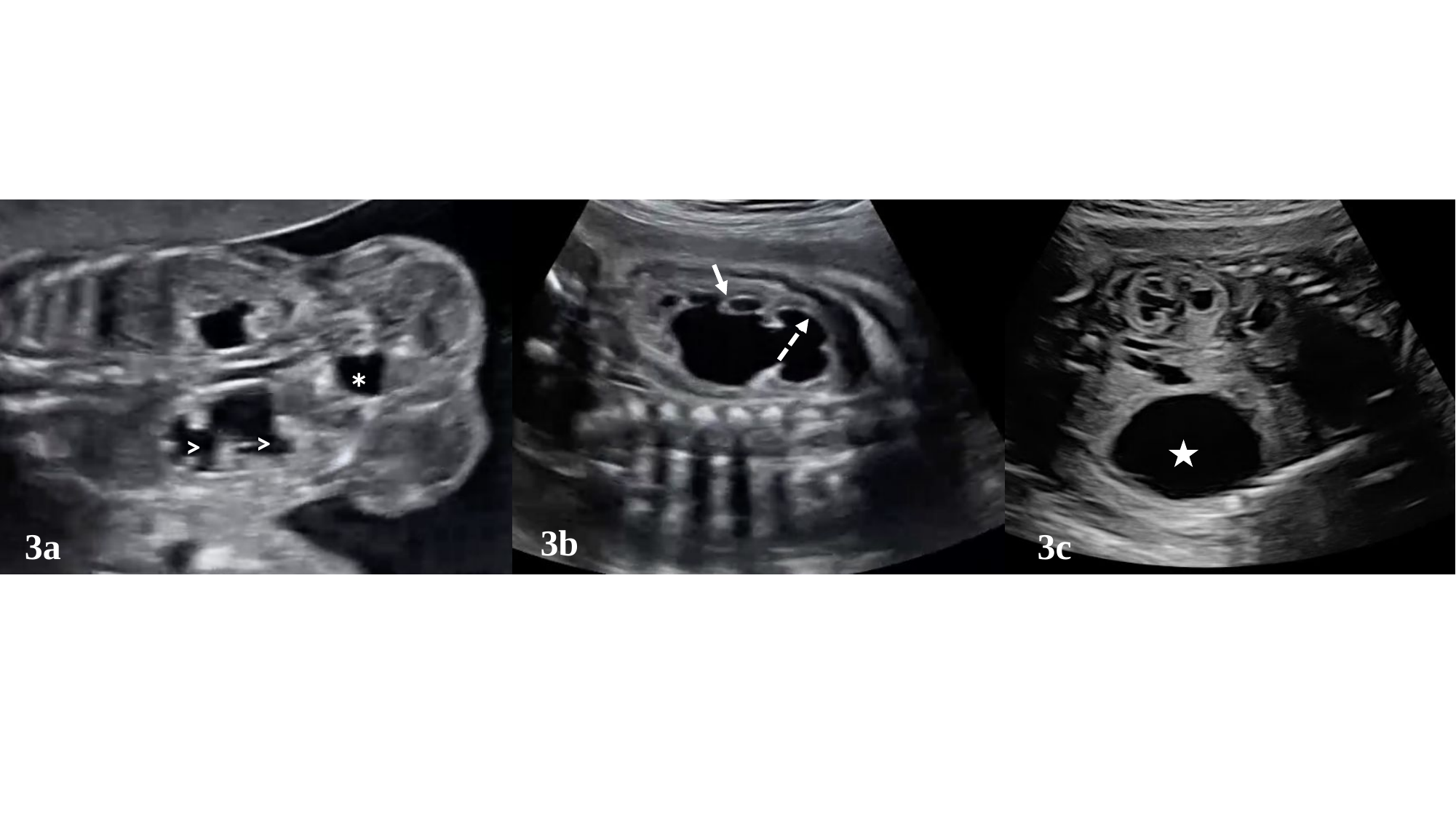

*
>
>
3b
3a
3c
1a
1b
1c
1a

## Slide 4
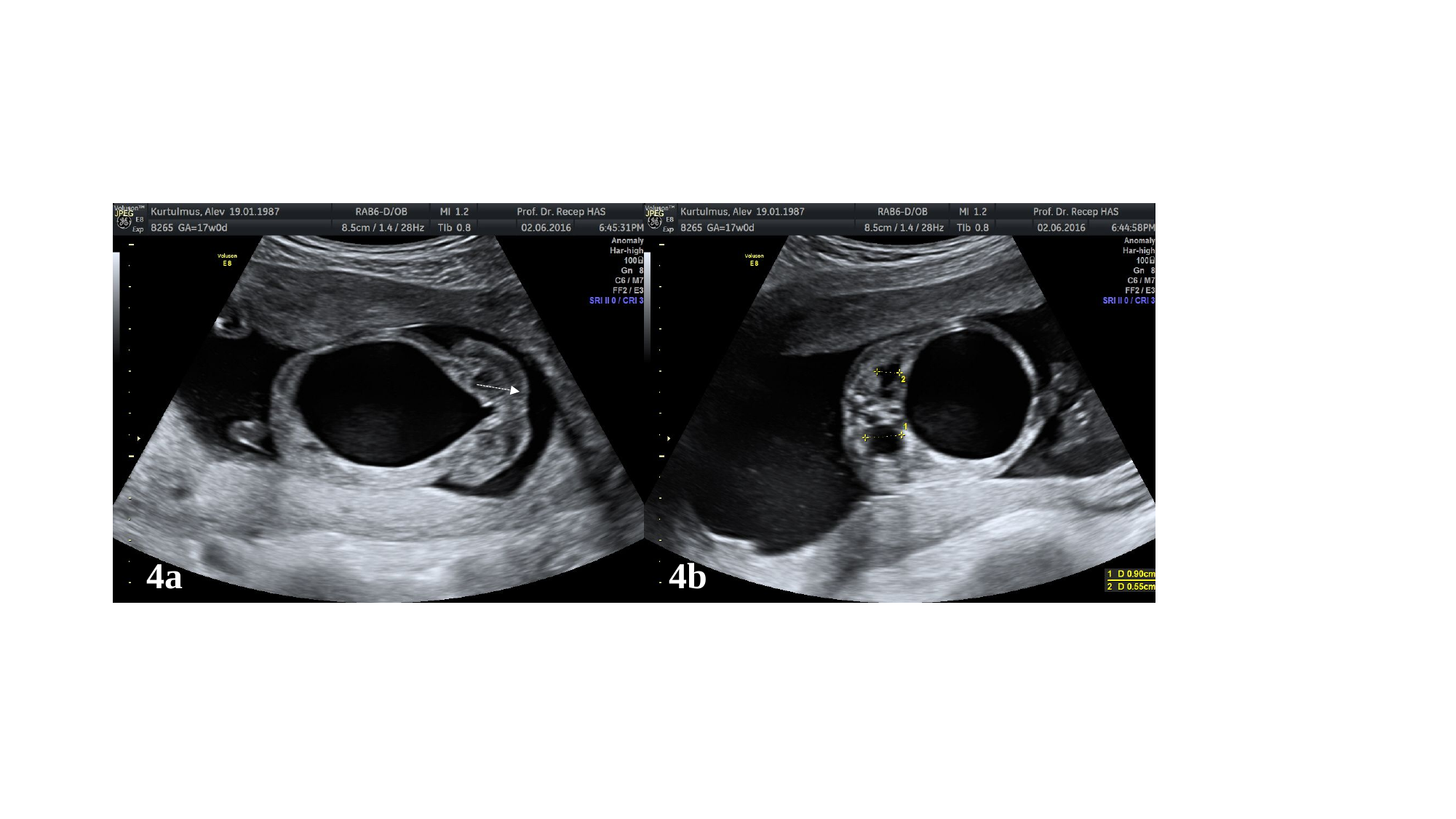

4b
4a

## Slide 5
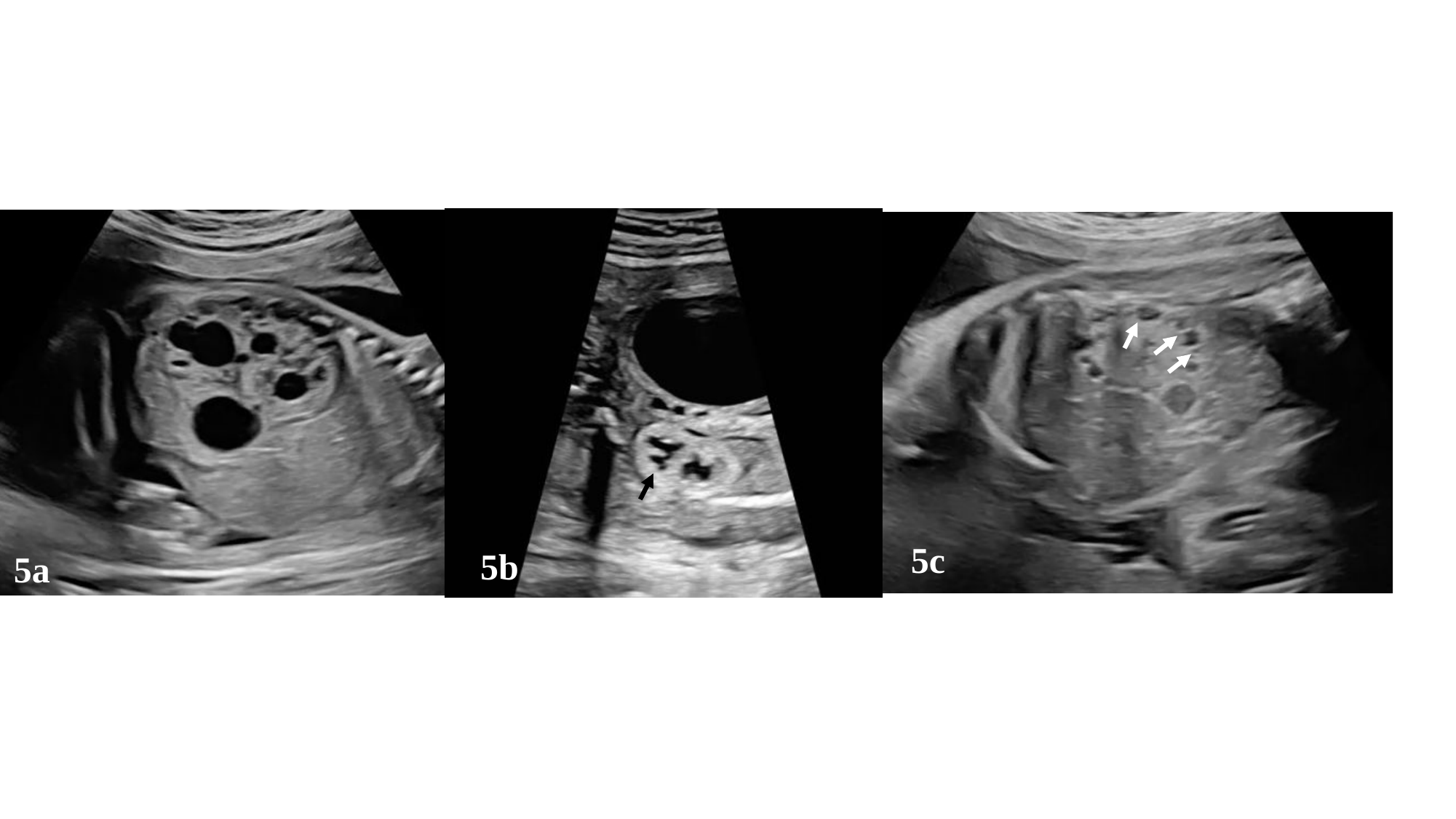

1a
1b
1c
5c
5b
5a

## Slide 6
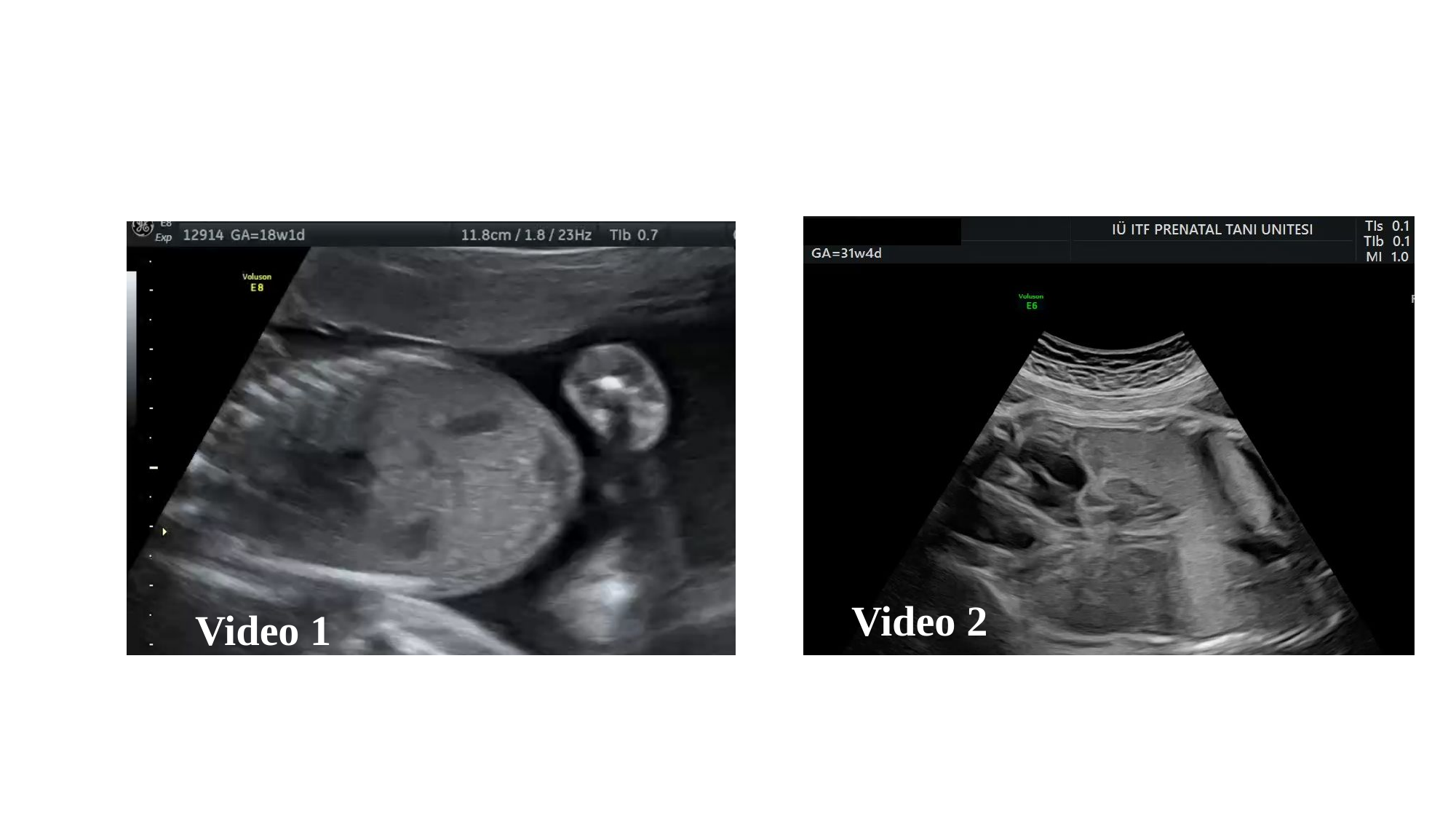

Video 2
Video 1

Supplement: Supplementary file 1 [file Presentation_1.PPTX]
